# 9th International Multithematic Scientific Bio-Medical Congress (IMBMC), Nicosia, Cyprus, 2021

**DOI:** 10.1038/s41419-022-04780-2

**Published:** 2022-04-06

**Authors:** Michael Luster, Karen Bang Strand, Anastasis Stephanou, Petros Agathaggelou, Ioannis Patrikios

**Affiliations:** School of Medicine, European University Cyprus, Nicosia, Cyprus

**Keywords:** Biomarkers, Biochemistry

The 9th International Multithematic Bio-Medical Congress 2021, “Bio-Medical Scientific Cyprus (BSC)”, was organized by the European University Cyprus (EUC), School of Medicine, Nicosia, Cyprus, with Cyprus Medical Association (CyMA) as a co-organizer and under the auspices of the Cyprus Ministry of Health and the Cyprus Biological Society (CBS). BSC is an internationally recognized annual event that was first founded and established by Professor Dr. Ioannis Patrikios, the Deputy Dean and Faculty member of the School of Medicine at EUC.

As the keynote speaker, Professor Dr. George Chrousos, *Professor of Pediatrics and Endocrinology Emeritus, UNESCO Chair on Adolescent Health Care, National and Kapodistrian University of Athens*, spoke about Greek Medical Philosophy and the Roots of Modern Medicine.

In tying the connection between pre-Hippocratic, Hippocratic, the resulting Hellenistic and modern Western medical thought and philosophy, Dr. Chrousos illustrated and illuminated the ever-changing and yet true-to-its-roots nature of healing and medicine. Tools and techniques may change with scientific advancements, but the Pythagorean principle of harmony remains a baseline consideration, even today as he mentioned with emphasis.

In “The Future of Cardiology,” Professor Dr. Thomas F. Lüscher, *Center for Molecular Cardiology, University of Zurich, Switzerland*, spoke about the history, present, and future of Cardiology as a discipline and field of research. Focusing on atherosclerosis, the major underlying cause of cardiovascular disease (CVD), he described recent research showing that life-long low LDL-C reduced the chances of major cardiovascular events (MACE) by up to 80 percent, followed by a preview into the capabilities and promises of CRISPR-Cas9 targeted therapies.

Dr. John P.A. Ioannidis, *Epidemiology and Population Health at Stanford University*, discussed his learnings from the epidemiology of COVID-19 over the past two years. Analyzing the success of various measures, he proposed a “road forward” into an endemic state of the viral outbreak and highlighted challenges and changes along the way. Furthermore, he discussed the expectation of the COVID-19 Pandemic epidemiological status to become endemic in early 2022.

Following on the theme of COVID-19, Professor Dr. Sotirios Tsiodras, *National and Kapodistrian University of Athens Medical School*, elaborated further on the challenges faced and ahead in the scientific approach towards the pandemic. He suggested denser monitoring of viral strains and variants, a much improved globally equitable and equal availability of vaccines and critical care technology, and better science communication within the community and towards the public.

Nadir Arber, MD, Shiran Shapira PhD, *Health Promotion Center and Integrated Cancer Prevention Center, Tel Aviv* and Tsiodras Sotirios MD, PhD, *Attikon University Hospital, National and Kapodistrian University of Athens Medical School* presented novel research and clinical trials in EXO-CD24, exosomes enriched with the immune-checkpoint-protein, CD24, as a therapeutic agent against virus-induced hyper-inflammation and ARDS.

The prevention of childhood acute leukemia was the focus of a talk by Professor Dr. Arndt Borkhardt, *Pediatric Oncology,-Hematology and Clinical Immunology, University Hospital, Heinrich-Heine-University, Düsseldorf, Germany*. He introduced a model of trained immunity in B-cell precursor acute lymphoblastic leukemia (BCP-ALL). Experimental evidence suggests that a major driver of conversion from the preleukemic to the leukemic state is exposure to immune challenges, and Dr. Borkhardt introduced the concept of “trained immunity” into existing models of childhood BCP-ALL and suggest future avenues toward leukemia prevention

Professor Dr. Stuart Ralston, *The University of Edinburgh* spoke about recent developments in Paget’s disease of bone, and the use of zoledronic acid, a bisphosphonate, in the treatment of associated pain. He also warned, that there is currently no evidence for the use of prophylactic bisphosphonate therapy aimed at normalizing bone turnover, pending further research.

“Inhibition of Transglutaminase type 2 prevents hepatocellular carcinoma development” was the title of the presentation by Professor Dr. Mauro Piacentini, *Department of Biology, University of Rome ‘Tor Vergata’, Rome, Italy*. In it, he outlined novel research showing the key role of Transglutaminase type 2 (TG2) in development of Hepatocellular carcinoma (HCC) and the efficacy of cysteamine (a TG2 inhibitor) in drastically reducing HCC development. In addition, he outlined some of the molecular mechanisms that are controlled by the enzyme and could represent further targets to develop a new therapeutic approach for the treatment of HCC.

Speaking on behalf of the *VACCELERATE Consortium’s EU-COVAT-1 AGED, EU-COVAT-2 BOOSTAVAC and EU-COVAT PAED study groups*, Oliver A. Cornely explained the group’s recent and upcoming clinical trials, their setup and management, and previous outcomes as well as study foci for two upcoming clinical trials in the efficacy of a third (booster) dose in adults aged 75 or older, and reduced booster doses in adolescents and children.

The surgery, cognitive preservation complications, and outcome of low-grade gliomas was the topic of a talk by Professor Dr. Zvi Ram, *Chairman of the Department of Neurosurgery at Tel Aviv Medical Center*. He outlined his current research and chanced a look into the future of translational imaging-guided low-impact surgeries, the use of immunotherapeutics in malignant brain tumors, and other low-impact approaches.

Dr. George K. Andrikopoulos, *Director of the 1st department of Cardiology and the department of Electrophysiology and Pacing, Henry Dunant Hospital, Athens, Greece* revisited the topic of atrial fibrillation and spoke about new ideas, novel concepts, and recent developments in the treatment and management thereof. He stressed the roles of early diagnosis, early treatment and early restoration of sinus rhythm in case of AF relapse, and looked ahead, predicting an end to “incurable arrhythmia” by the hand of evidence-based medicine.

Risk assessment models for venous thromboembolism (VTE) in ambulatory patients with cancer was the focus of a talk by Associate Professor Dr. Grigoris Gerotziafas, *Professor of Hematology, Faculty of Medicine Sorbonne University*. Beginning with the publication of the original Khorana Risk Score (KRS), a new generation of KRS based scoring models has arisen, of which CATS/MICA offers the simplest and most user friendly approach while COMPASS-CAT introduced the more holistic inclusion of patient comorbidities. Concluding his talk, Dr. Gerotziafas stressed the need for hypercoagulability biomarker inclusion in the development of a full precision medicine model, aided by machine learning and AI methodologies.

Associate Professor Dr. Giuseppe S. Sica, *Associate Professor of Surgery at the University of Rome Tor Vergata, Italy*, gave an update on colon and rectal cancer treatments and stressed that it was time to develop better preventative, diagnostic, and treatment models.

Likewise, Dr. Dimitrios Kyparissopoulos, *Honorary Consultant Thoracic Surgeon at Royal Brompton & Harefield NHS Foundation Trust*, spoke about the latest trends in surgical treatment of lung cancer in the robotic DaVinci era, spoke about the advantages and disadvantages of this approach, and screened videos demonstrating the procedure. The talk was rounded off by personal experience reports and outcome demonstrations.

Professor Dr. Kyriakos E. Kypreos, *Chairman of the Pharmacology laboratory of the Department of Medicine at the University of Patras School of Health Sciences, Patra, Greece*, returned to the question of high-density lipoprotein cholesterol (HDL-C) levels and the risk for CVD. His presentation outlined current research and novel preventative methods in this field.

The role of p63 in the female germline fidelity and related cancers was the focus of Professor Dr. Gerry Melino, *Department Experimental Medicine, TOR, University of Rome “Tor Vergata*”. His demonstrated research showed, that the p63 C-terminus is essential in TAp63α-expressing primary oocytes to control cell death in vivo, expanding the current understanding of human Primary Ovarian Insufficiency (POI). He suggested a new use for kinase inhibitors, aimed at preserving oocytes of the follicle reserve during chemotherapeutic treatments.

Professor Dr. Stavros Konstantinides, *Professor for Clinical Trials and Medical Director of the Center for Thrombosis and Hemostasis (CTH), University of Mainz, Germany* spoke about the future of anti-thrombotic treatments. He investigated the use of monoclonal antibodies as blockers and deactivants of FXIa, as well as the use of small molecule inhibitors in replacement of direct oral anti-Xa and antithrombin agents. Further research will be needed, he stressed.

These meeting notes and report summarize the major scientific findings from the aforementioned presenters. Unpublished data may also be included.

